# Curcumin Stimulates the Antioxidant Mechanisms in Mouse Skin Exposed to Fractionated γ-Irradiation

**DOI:** 10.3390/antiox4010025

**Published:** 2015-01-13

**Authors:** Ganesh Chandra Jagetia, Golgod Krishnamurthy Rajanikant

**Affiliations:** 1 Department of Zoology, Mizoram University, Aizawl-796 004, India; 2 School of Biotechnology, National Institute of Technology, Calicut-673 601, India; E-Mail: rajaniatrb@yahoo.com

**Keywords:** curcumin, mice, skin, irradiation, antioxidant enzymes, lipid peroxidation

## Abstract

Fractionated irradiation is one of the important radiotherapy regimens to treat different types of neoplasia. Despite of the immense therapeutic gains accrued by delivering fractionated irradiation to tumors, the radiation burden on skin increases significantly. Low doses of irradiation to skin adversely affect its molecular and metabolic status. The use of antioxidant/s may help to alleviate the radiation-induced changes in the skin and allow delivering a higher dose of radiation to attain better therapeutic gains. Curcumin is an antioxidant and a free radical scavenging dietary supplement, commonly used as a flavoring agent in curries. Therefore, the effect of 100 mg/kg body weight curcumin was studied on the antioxidant status of mice skin exposed to a total dose of 10, 20 and 40 Gy γ-radiation below the rib cage delivered as a single fraction of 2 Gy per day for 5, 10 or 20 days. Skin biopsies from both the curcumin treated or untreated irradiated groups were collected for the biochemical estimations at various post-irradiation times. The irradiation of animals caused a dose dependent decline in the glutathione concentration, glutathione peroxidase, and superoxide dismutase activities and increased the lipid peroxidation in the irradiated skin. Curcumin treatment before irradiation resulted in a significant rise in the glutathione concentration and activities of both the glutathione peroxidase and superoxide dismutase enzymes in mouse skin, whereas lipid peroxidation declined significantly. The present study indicates that curcumin treatment increased the antioxidant status of mouse exposed to different doses of fractionated γ-radiation.

## 1. Introduction

Skin is the outer most covering of the body, which protects the body against the invading pathogens, evaporation, and degradation of folate. It also insulates the body against the external environment, regulates body temperature, synthesizes Vitamin D, and provides a waterproof covering to the body [1]. Ionizing radiation is an important treatment modality in the management of various neoplastic disorders, where cancer patients are usually exposed to 20–30 fractions of 1.5–2 Gy each spread over a period of 5–6 weeks so as to spare the normal tissues and at the same time increase the radiotoxicity of tumors. Despite immense therapeutic benefit, the normal skin invariably suffers from the cytotoxic effect of radiation. According to the classification of the detrimental somatic effects of ionizing radiation, the radiation-induced damage to the skin can be both stochastic and non-stochastic [2].

Consistent with this view, ionizing radiation interacts with skin and undoubtedly damages it by direct or indirect processes by generating numerous radiolytic reactive oxygen products including ^•^OH, H^•^, O_2_^•−^, HOO^•^ free radicals and H_2_O_2_ in aqueous milieu of the skin cells [3]. A plethora of defense mechanisms exists in skin [4,5,6,7] that can deal with the onslaught of reactive oxygen species (ROS) under normal conditions; however, an additional oxidative stress generated after exposure to ionizing radiation may not be efficiently handled by it [8]. The management of skin reactions after radiotherapy is a challenging task after radiotherapy as –the low level of fractionated irradiation has been linked to the induction of basal and squamous cell skin carcinomas [9]. It is also associated with radiation-induced oedema, erythema, pigmentation, fibrosis, ulceration, pain, warmth, burning, itching of the skin, dermatitis and dry and moist desquamation [10,11]. Skin irradiation to low doses of radiation has been found to alter its metabolic profile [12]. Some of the general events associated with early phase of oxidative stress response in the skin are depletion of endogenous intra- and inter-cellular antioxidants, enhancement of intracellular lipid peroxidation levels and the induction of specific signal transduction pathways that can modulate inflammatory, immune suppressive or apoptotic processes in the skin [13,14,15]. It is necessary to counter the additional oxidative stress induced by ionizing radiation by exogenous supply of antioxidants, which could act as safe and effective skin protectants in the irradiated skin. The introduction of exogenous antioxidants that scavenge ROS and restore normal redox state may be beneficial in such a situation [16,17,18,19]. Application of such antioxidants to protect normal tissues like skin from radiation injury might allow one to increase the radiation dose to the tumor thus increasing the probability of better tumor control in clinics.

The polyphenol curcumin or diferuloylmethane (1,7-bis (4-hydroxy-3-methoxy-phenyl) hepta-1, 6-diene-3, 5-dione) is one of the most active molecules present in the turmeric rhizome (family: Zingiberaceae) that imparts the characteristic yellow color to curry powder, the turmeric [20]. Curcumin is widely used as a spice, dietary pigment, and as an Indian folk medicine for the treatment of various illnesses including cough, common cold, sore throat and healing of wounds. Traditional medicinal practitioners prescribe turmeric to treat rheumatism, body pain, skin diseases, intestinal worms, diarrhea, intermittent, fevers, hepatic disorders, biliousness, urinary discharges, dyspepsia, inflammations, constipation, leucoderma, amenorrhea, and colic. Turmeric is an emmenagogue, diuretic, carminative, and it is applied externally against bruises, pains, sprains, boils, swellings, and skin diseases [21]. In China, turmeric is used to treat angina pectoris, stomachache, postpartum abdominal pain, and gallstones [22]. Current traditional Indian medicine uses turmeric to treat biliary disorders, anorexia, cough, diabetic wounds, hepatic disorders, rheumatism, and sinusitis. This nonnutritive curry flavoring phytochemical is pharmacologically safe, since it has been used as a dietary spice up to a dose of 100 mg/day, for centuries [23,24]. Humans can tolerate a dose as high as 12 g/day of curcumin without any untoward toxic side effects [25], which reaffirms its nontoxic nature.

Curcumin and related compounds have the ability to induce glutathione-*S*-transferase, inhibit free radical generation and act as a free radical scavengers and antioxidants, inhibiting lipid peroxidation [26,27,28,29,30]. Because ROS have been known to play an important role in radiation-induced skin injury, one might expect that curcumin, as an antioxidant, radioprotector and free radical scavenger, would protect skin from radiation damage by increasing the antioxidant status of the skin. Earlier studies have revealed that curcumin pretreatment protected mouse against the deep dermal irradiated wound after whole body, hemi-body and fractionated irradiation [19,31,32,33]. Recently, curcumin has been reported to enhance re-epithelialization in CO_2_ laser-induced incision wounds in mice [34]. Curcumin is a dietary supplement and is not synthesized in the body. Therefore, the present study was carried out to obtain an insight into the effect of curcumin on antioxidant status and lipid peroxidation in the mouse skin exposed to 10, 20 and 40 Gy of fractionated γ- radiation.

## 2. Materials and Methods

The animal care and handling were carried out according to the guidelines issued by the World Health Organization, Geneva, Switzerland and the INSA (Indian National Science Academy, New Delhi). Usually, eight to 10-week old male and female (1:1 ratio) Swiss albino mice weighing 30–36 g were selected from an inbred colony maintained under the controlled conditions of temperature (23 ± 2 °C), humidity (50% ± 5%) and light (12 h of light and dark cycle, respectively). The animals were given sterile food and water throughout the experiment. The food consisted of 50% cracked wheat, 40% Bengal gram, 4% milk powder, 4% yeast powder, 0.75% sesame oil, 0.25% cod liver oil, and 1% salt. Four animals were housed in a sterile polypropylene cage containing sterile paddy husk (procured locally) as bedding during the experimentation. The study had been approved by the institutional animal ethical committee of Kasturba Medical College, Manipal, India, where the whole study was carried out.

### 2.1. Preparation of Drug and Mode of Administration

Curcumin (Catalog No: C1386) was procured from Sigma Chemical Co. (St. Louis, MO, USA). The required amount of curcumin was suspended in 0.5% carboxymethylcellulose (CMC) and the animals were administered with 0.01 mL/g body weight of CMC or curcumin (CUM) orally.

### 2.2. Experimental Protocol

The animals were divided into the following groups:

***CMC + Sham-Irradiation (SIR):*** The animals of this group received 0.01 mL/g body weight of 0.5% CMC daily, consecutively for 5, 10 and 20 days, respectively, before sham-irradiation.

***CUM + SIR:*** The animals of this group received 100 mg/kg body weight of curcumin daily, consecutively for 5, 10 and 20 days, respectively, before sham-irradiation.

***CMC + irradiation (IR):*** The animals of this group received 0.01 mL/g body weight of 0.5% CMC once daily before exposure to 2 Gy/day consecutively for 5, 10 or 20 days, respectively.

***CUM + IR:*** The animals of this group received 100 mg/kg body weight of curcumin once daily before exposure to 2 Gy/day consecutively for 5, 10 or 20 days, respectively [19].

### 2.3. Irradiation

One hour after each administration of CMC or curcumin, each animal was placed into a specially designed well-ventilated acrylic restrainer and the lower half of the animal body (below the rib cage) was exposed to 0 or 2 Gy once daily, delivered at a dose rate of 1.35 Gy/min from a ^60^Co Teletherapy source (Theratron, Atomic Energy Agency, Ontario, Canada). Treatments were given once daily for 5, 10 or 20 days resulting in a cumulative dose of 10 Gy (a total of 5 fractions of 2 Gy each), 20 Gy (a total of 10 fractions of 2 Gy each) or 40 Gy (a total of 20 fractions of 2 Gy each), respectively. A two-day gap was allowed after the delivery of fifth, 10th and 15th fractions of radiation for mice receiving total doses of 20 or 40 Gy.

### 2.4. Preparation of Animals

The hairs were depilated from the lower half of the dorsum of the animals before the last exposure. Skin biopsies from each dose of all groups were collected at 0, 1.5, 3, 6, 12, 24, 48, 72 and 144 h after the last exposure. The skin was freed from *panniculus carnosus* and flash frozen in the liquid nitrogen. The skin was weighed and homogenized in the phosphate buffered saline. Four animals were used for each irradiation dose at each time point in all concurrent groups.

### 2.5. Biochemical Parameters

#### 2.5.1. Glutathione (GSH)

GSH content was measured by the method of Moron *et al*., [35]. Briefly, proteins were precipitated by 25% TCA, centrifuged and the supernatant was collected. The supernatant was mixed with 0.2 mol sodium phosphate buffer pH 8.0 and 0.06 mmol DTNB and incubated for 10 min at room temperature. The absorbance of the sample/s was read against the blank at 412 nm in a UV-Visible Spectrophotometer (Shimadzu UV-260, Shimadzu Corp., Tokyo, Japan) and the GSH concentration has been calculated from the standard curve.

#### 2.5.2. Glutathione Peroxidase (GSHPx)

GSHPx activity was estimated by the method of Sazuka *et al*., [36]. Briefly, 100 μL homogenate was mixed with 200 μL each of EDTA, sodium azide, GSH, H_2_O_2_ and 400 μL buffer. The reaction mixture was incubated at 37 °C for 10 min followed by the addition of 10% TCA. After centrifugation, the supernatant was collected and mixed with 3 mL disodium hydrogen phosphate and 1000 μL DTNB. The absorbance of the sample/s was recorded against the blank at 412 nm using UV-Visible Spectrophotometer. The activity has been expressed as μmol GSH/mg protein.

#### 2.5.3. Superoxide dismutase (SOD) 

The SOD activity was measured by the method of Fried [37]. Briefly, 900 μL buffer was mixed with 100 μL each of tissue homogenate (T), nitroblue tetrazolium (NBT), phenazine methosulphate and NADH. The control (C) consisted of all the reagents except the homogenate, whereas the blank (B) consisted of buffer and the homogenate without any reagents. The absorbance of T, C and B was measured at 560 nm using a UV-Visible Spectrophotometer and the enzyme activity has been expressed in units (1 U = 50% inhibition of NBT reduction).

#### 2.5.4. Lipid Peroxidation (LOO)

LOO was measured by the method of Beuege and Aust [38]. Briefly, the tissue homogenate was mixed with TCA-TBA-HCl solution, butylated hydroxytoluene (BHT, 3.5 mmol, 0.1 mL) and diethylenetriaminopentaacetic acid (DETAPAC, 70 μmol, 0.1 mL). The mixture was heated for 15 min in a boiling water bath. After centrifugation the absorbance was recorded at 535 nm using a UV-Visible Spectrophotometer. The lipid peroxidation has been expressed as MDA in nmol per mg protein.

#### 2.5.5. Estimation of Protein

Protein contents were measured by the method of Lowry *et al*., (1951) using bovine serum albumin (BSA) as a standard.

### 2.6. Analysis of Data

The statistical significance between various groups was determined using student’s *t*-test. Solo 4 statistical package (BMDP Statistical Software, Inc., Los Angeles, CA, USA) was used for data analysis. All the data are expressed as mean ± SEM (standard error of the mean).

## 3. Results

### 3.1. Glutathione (GSH)

**Figure 1:**
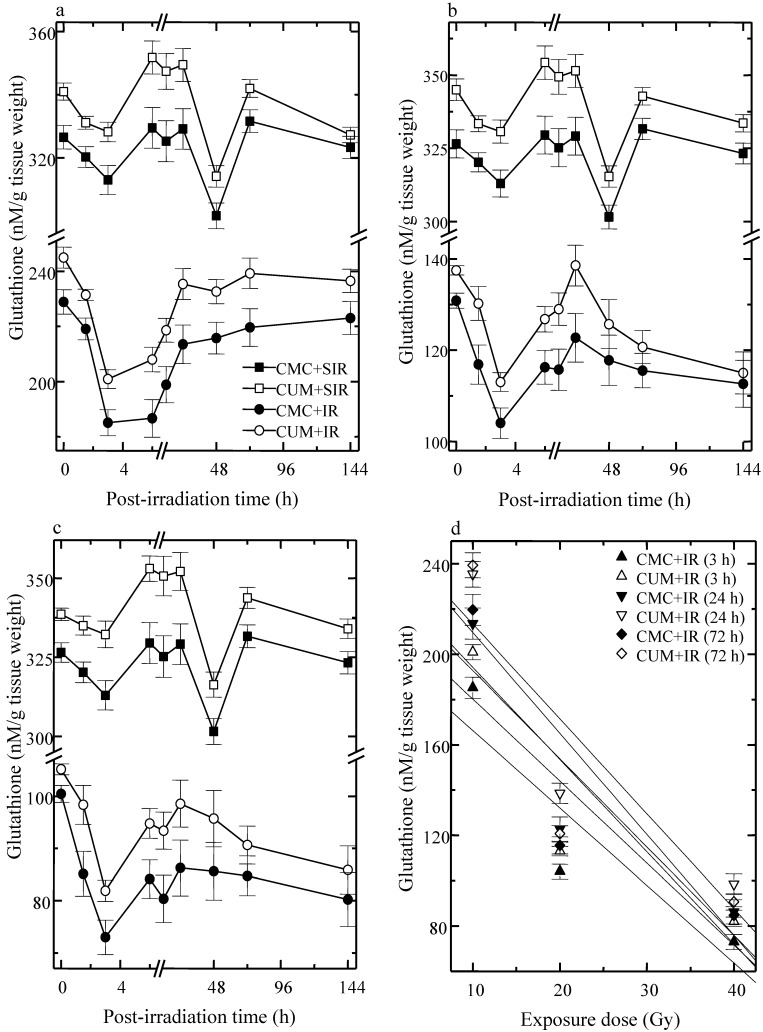
Effect of curcumin on glutathione concentration in the skin of mice exposed to fractionated doses of γ-radiation. (**a**) 10 Gy (**b**) 20 Gy (**c**) 40 Gy and (**d**) dose response relationship.

GSH content declined immediately after wound creation up to 3 h and then elevated at 6 h post-irradiation in CMC + SIR group. A second decrement in GSH level was observed at 48 h post-irradiation. However, treatment of mice with curcumin before sham-irradiation resulted in a significant (*p* < 0.05) elevation in the GSH contents for all post-irradiation times. The irradiation of animals to various doses of fractionated γ-radiation resulted in a drastic but dose dependent decline in the GSH contents when compared with CMC + SIR group and a nadir was reached at 3 and 6 h post-irradiation at all exposure doses in both the CMC + IR and CUM+IR groups (Figure 1). Curcumin treatment prior to irradiation caused a significant elevation (*p* < 0.05, up to 24 h post-irradiation for 40 Gy) in GSH concentration when compared with the concurrent CMC + IR group. However, normal levels could not be restored even by 144 h post-irradiation in both CMC + IR and CUM + IR groups (Figure 1).

### 3.2. Glutathione Peroxidase (GSHPx)

**Figure 2:**
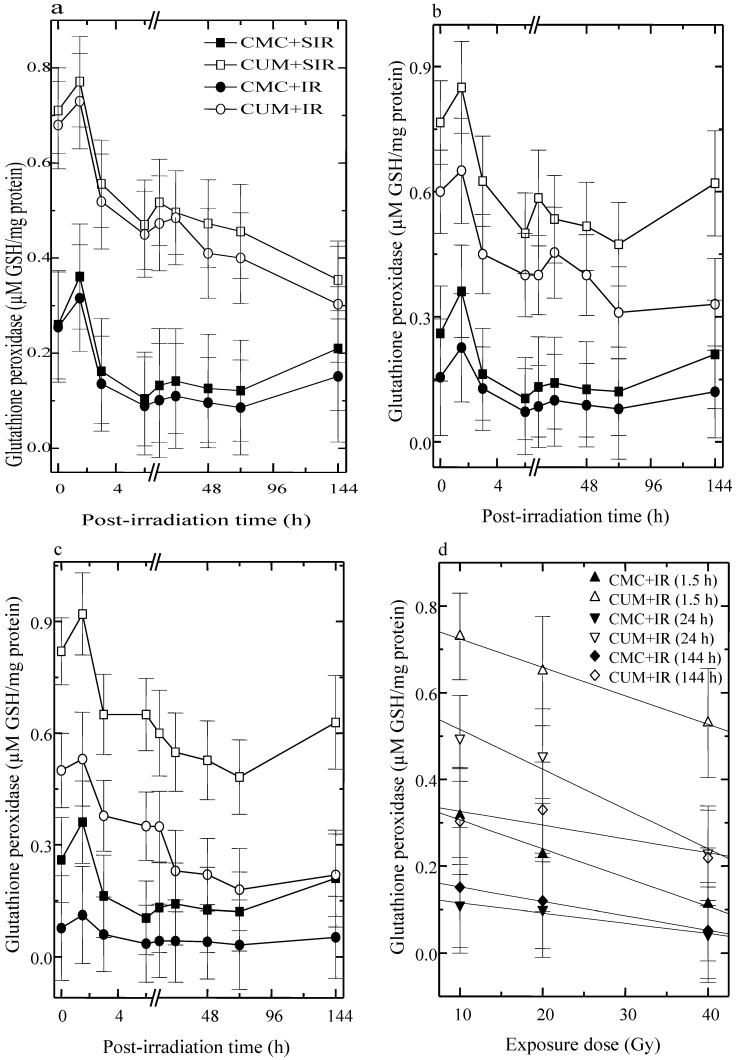
Effect of curcumin on glutathione peroxidase activity in the skin of mice exposed to different doses of fractionated γ-radiation. (**a**) 10 Gy (**b**) 20 Gy (**c**) 40 Gy and (**d**) dose response relationship.

The activity of GSHPx fluctuated with time in CMC + SIR group and a highest elevation was observed at 1.5 h post-irradiation. A second rise in GSHPx activity was observed at 24 h, which remained unaltered up to 72 h post-irradiation. Curcumin administration before sham-irradiation resulted in a significant elevation (*p* < 0.05 up to 72 h post-irradiation for five fractions of curcumin) in the GSHPx activity, when compared with CMC + SIR groups. The irradiation of animals to different doses of fractionated γ-radiation resulted in a dose dependent alleviation in the GSHPx activity in both the CMC + IR and CUM + IR groups when compared with the respective sham-irradiation groups (Figure 2). The pattern of fluctuation in GSHPx activity after exposure to different doses of fractionated γ-radiation was similar to that of sham-irradiation groups. The administration of curcumin before exposure to various doses of γ-radiation caused a drastic rise in the GSHPx activity and this increase was highest at 1.5 h (*p* < 0.02) followed by 24 h (*p* < 0.03) and 144 h post-irradiation in CUM + IR groups (Figure 2). However, spontaneous levels could not be restored even at 144 h post-irradiation.

### 3.3. Superoxide Dismutase (SOD)

**Figure 3:**
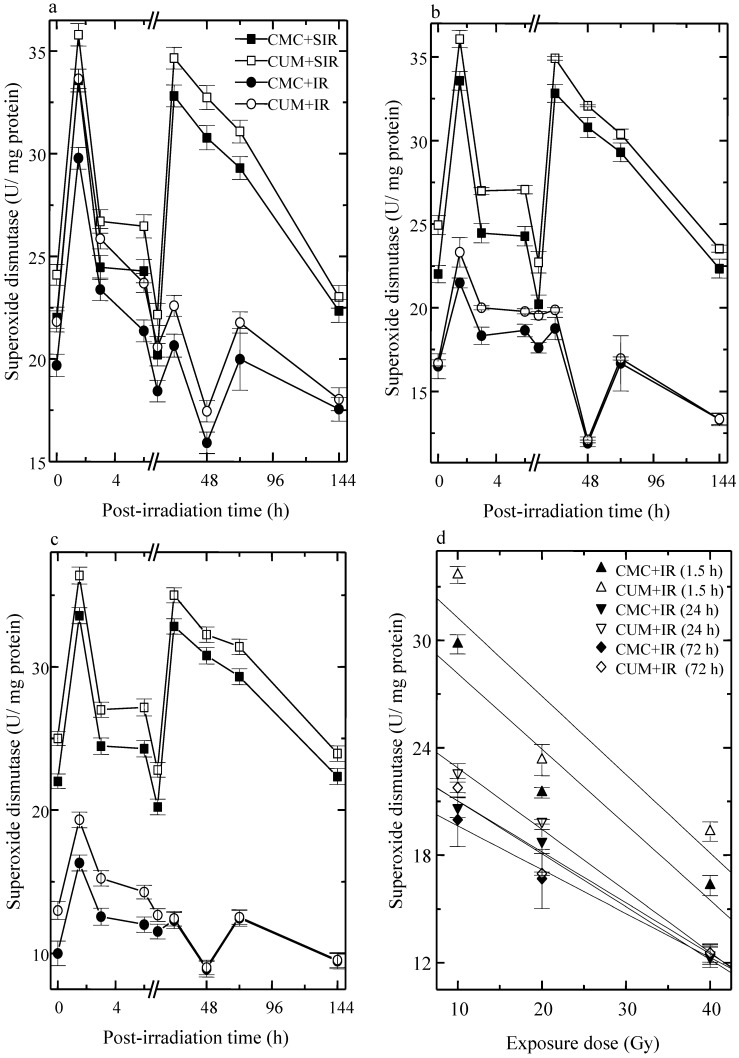
Effect of curcumin on superoxide dismutase activity in the skin of mice exposed to fractionated doses of fractionated γ-radiation. (**a**) 10 Gy (**b**) 20 Gy (**c**) 40 Gy and (**d**) dose response relationship.

The analysis of SOD activity showed a peak level at 1.5 that declined thereafter and attained a nadir at 48 h post-irradiation in CMC + SIR group. Treatment of mice with curcumin before sham-irradiation elevated the SOD activity significantly when compared with the CMC + SIR group. The exposure of mice to different doses of fractionated radiation resulted in a significant and dose dependent reduction in the SOD activity when compared with the CMC+SIR group, whereas curcumin treatment before exposure to various doses of fractionated radiation caused a significant augmentation (*p* < 0.05, up to 72 h post-irradiation for 10 Gy) in the SOD activity in CUM+IR group when compared with concurrent CMC + IR group (Figure 3). This elevation was approximately two-fold greater at 1.5 h post-irradiation for all exposure doses. The second rise in SOD activity was discernible at 24 h (*p* < 0.05) post-irradiation for 10, 20 and 40 Gy in CUM + IR group. The pattern of SOD activity in CUM + IR group was similar to that of CMC + IR group (Figure 3).

### 3.4. Lipid Peroxidation (LOO)

**Figure 4:**
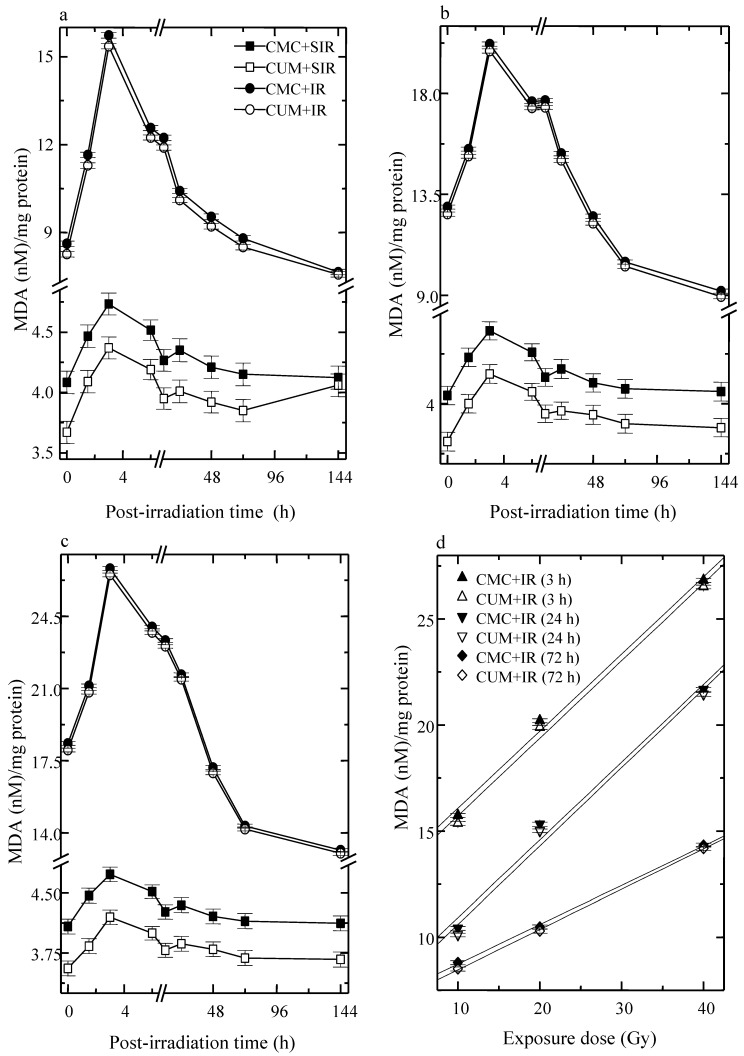
Effect of curcumin on lipid peroxidation level in the skin of mice exposed to fractionated doses of γ-radiation. (**a**) 10 Gy (**b**) 20 Gy (**c**) 40 Gy and (**d**) dose response relationship.

Infliction of excision wound caused an elevation in LOO in CMC + SIR group. LOO fluctuated with time in both CMC + SIR and CUM + SIR groups, where a highest elevation was observed at 3 h post-irradiation and a gradual decline thereafter. Curcumin treatment resulted in a significant (*p* < 0.05) decline in the spontaneous levels of LOO. Induction of LOO increased with an increase in irradiation dose (maximum for 40 Gy) as well as with assay time in both the CMC + IR and CUM + IR groups and a peak LOO was reached at 3 h post-irradiation. Thereafter, LOO declined steadily and reached a nadir at 144 h post-exposure (Figure 4). Curcumin pretreatment afforded a significant (*p* < 0.05, up to 24 h post-irradiation for 40 Gy) protection against the radiation-induced lipid peroxidation. However, the normal LOO levels could not be restored even by 144 h post-irradiation (Figure 4).

## 4. Discussion

In spite of the immense therapeutic gains produced by the fractionated irradiation regimens, radiation burden on the skin increases significantly. To a large extent, radiation injury to living cells is mediated by the generation of oxygen derived free radicals and hydrogen peroxide. Consequently, protection of skin by exogenous supply of antioxidants that scavenge ROS and restore normal redox state may allow increasing the radiation dose for better therapeutic gains. Curcumin—an active polyphenolic compound of turmeric, and a well-known culinary agent and curry spice, extensively used as a food additive to impart yellow color—was investigated for its efficacy to modulate radiation-induced alleviation of the antioxidant status in the skin of mice exposed to various doses of fractionated γ-radiation.

A direct quantitative comparison of the results of the present study to those reported by other investigators may not be possible, because of the use of different experimental protocols and conditions of assays (pH, temperature, reagent concentration) and different unit definitions. Nevertheless, results of the present investigation are qualitatively in good agreement with other studies, where similar effects have been reported [18,39]. Ionizing radiation induces ROS and lipid peroxidation that subsequently depletes antioxidant status in the skin [3,18]. Fractionated irradiation of mice skin caused a drastic decline in the GSH concentration and GSHPx and SOD, activities. A similar reduction in GSH concentration and GSHPx and superoxide dismutase activities has been reported earlier in mice skin exposed to fractionated γ-radiation [18]. A decline in the erythrocyte GSHPx and SOD activity has been observed in female rats during radiotherapy [40]. Whole body irradiation has been reported to reduce GSH concentration and the activities of GSHPx and SOD in the mouse liver earlier [41]. Ionizing radiation induces lipid peroxidation and a similar effect has been observed in the present study where, exposure of mice to various doses of fractionated gamma radiation resulted in an increase in lipid peroxidation and decreased the antioxidant status of the skin. Irradiation has been reported to increase the lipid peroxidation in mouse liver, stomach, small intestine and kidney and human peripheral blood lymphocytes earlier [41,42,43,44,45]. Curcumin pretreatment afforded protection against radiation-induced elevation in lipid peroxidation and arrested the radiation-induced decline in GSH concentration and GSHPx, and SOD activity. A similar observation has been reported earlier in mice skin treated with ascorbic acid before fractionated irradiation [18]. Curcumin has been reported to protect mouse liver against the radiation-induced decline in GSH concentration and GSHPx, and SOD activity and lipid peroxidation [45]. Similarly, ginger, bael, naringin and mangiferin have been reported to elevate the GSH concentration and GSHPx, and SOD activity and reduce radiation-induced lipid peroxidation [42,43,44].

The redox state of the cell is primarily a consequence of the precise balance between the levels of ROS and endogenous thiol buffers present in the cell, such as glutathione and thioredoxin, which protect cells from oxidative damage. Dramatic elevation of ROS, exceeding compensatory changes in the level of the endogenous thiol buffers, may result in the sustained activation of signaling pathways and expression of genes that induce apoptosis in affected cells [46]. Nuclear factor-(erythroid derived 2)-related factor-2 (Nrf2) resides in cytoplasm and translocates into nucleus to initiate antioxidant pathway [47]. Curcumin has been reported to upregulate Nrf2 gene in irradiated rat brain [48]. The upregulation of Nrf2 pathway may be involved in the rise in the GSH concentration, GSHPx and SOD activity after curcumin treatment in this study. Glutathione acts synergistically with other endogenous antioxidants and acts as a co-factor with the enzyme glutathione peroxidase to scavenge free radicals, and detoxify xenobiotics [49,50]. The depletion in GSH contents after exposure to γ-radiation may be due to the reaction of GSH with free radicals resulting in the formation of thiyl radicals that react to produce GSSG as reported earlier [51]. Depletion of glutathione *in vitro* and *in vivo* is known to cause inhibition of glutathione peroxidase activity and has been shown to increase lipid peroxidation [52]. A similar correlation between the depletion of GSH and elevation of LOO exists in the present investigation, which is consistent with the results of previous studies [18,53,54,55].

It is commonly accepted that SOD protects against the free radical damage by scavenging superoxide radical, converting, superoxide radical into H_2_O_2_ and prevents the formation of ^•^OH radical through superoxide driven Fenton reaction [56]. The H_2_O_2_ thus generated can be removed by catalase or GSHPx. However, radiation-induced decline in both SOD and GSHPx activity along with GSH promotes the formation of ^•^OH radicals, and initiation and propagation of LOO [57]. The increase in MDA levels and the concomitant decrease in antioxidant enzymes and GSH levels demonstrate the role of oxidative mechanisms in radiation-induced tissue damage. Glutathione peroxidase also protects the cell protein and membrane against oxidation [58]. The initial elevation of LOO levels seems to be due to the ROS-producing effect of radiation and the resultant formation of lipid peroxides from unsaturated fatty acids. The lower enzyme activities observed after irradiation were most likely due to the enzyme inactivating activity of ROS induced by radiation.

The mitigation of cytotoxic effect of ROS by curcumin may be due to the direct scavenging of free radicals and subsequent inhibition of oxygenation reaction as curcumin has been reported to be a good antioxidant, free radical scavenger and a radioprotective agent [27,59,60]. A similar free radical scavenging action of radiation-induced free radicals by curcumin seems to be operational in the present study. Curcumin may have lowered lipid peroxidation by maintaining the activities of antioxidant enzymes and glutathione at higher levels as these enzymes play an important role in the regulation of lipid peroxidation. This may have contributed to a rapid scavenging of radicals and consequently less damage to the skin. Curcumin has been reported to alleviate radiation-induced lipid peroxidation in the irradiated skin of mice [30]. It has been demonstrated that dietary supplementation of curcumin (2%, w/v) to male ddY mice for 30 days significantly increased activities of antioxidants and phase II-metabolizing enzymes in liver and kidney when compared to corresponding normal diet fed control mice [61]. Our earlier study has demonstrated that ascorbic acid treatment prior to irradiation significantly improved skin antioxidant status in mice after exposure to fractionated irradiation [18].

Our results suggest that oral administration of curcumin before irradiation significantly elevated the activity of glutathione concentration, glutathione peroxidase and superoxide dismutase activity and lowered the lipid peroxidation in mice skin exposed to various doses of fractionated γ-radiation. This activity of curcumin may be due to free radical scavenging and upregulation of Nrf2 expression. Our findings indicate that curcumin pretreatment may be one of the paradigms to reduce the effect of radiation on the skin of patients receiving radiotherapy for the treatment of cancer.
